# Field evaluation of the novel One Step Malaria Pf and Pf/Pv rapid diagnostic tests and the proportion of HRP-2 gene deletion identified on samples collected in the Pwani region, Tanzania

**DOI:** 10.1186/s42269-023-00992-4

**Published:** 2023-02-07

**Authors:** Zena E. Mwangonela, Young Ye, Qin Rachel, Hajirani M. Msuya, Tunu G. Mwamlima, Sarah S. Mswata, Prosper P. Chaki, Ester G. Kimaro, Clement N. Mweya, Maxmillian G. Mpina, Grace W. Mwangoka

**Affiliations:** 1 Ifakara Health Institute Bagamoyo Branch, P.O.Box 74, Bagamoyo, Tanzania; 2 The Nelson Mandela African Institution of Science and Technology, P.O.Box 447, Arusha, Tanzania; 3 InTec Products, Inc., 332 Xinguang Road Xinyang Industrial Area, Haicang, Xiamen City, 361022 China; 4 Univesity of Dar Es Salaam, Mbeya College of Health and Allied Science, P.O.Box 608, Mbeya, Tanzania

**Keywords:** Malaria, Rapid diagnostic test, Sensitivity, Specificity, HRP-2 gene deletion

## Abstract

**Background:**

Malaria rapid diagnostic tests (mRDTs) have played an important role in the early detection of clinical malaria in an endemic area. While several mRDTs are currently on the market, the availability of mRDTs with high sensitivity and specificity will merit the fight against malaria. We evaluated the field performance of a novel One Step Malaria (P.f/P.v) Tri-line and One Step Malaria (P.f) rapid test kits in Pwani, Tanzania.

**Methods:**

In a cross-sectional study conducted in Bagamoyo and Kibiti districts in Tanzania, symptomatic patients were tested using the SD BIOLINE, One Step Malaria (P.f/P.v) Tri-line and One Step Malaria (P.f) rapid test kits, microscope, and quantitative Polymerase Chain Reaction (qPCR). An additional qPCR assay was carried out to detect Histidine-Rich Protein 2 (HRP-2) gene deletion on mRDT negative but microscope and qPCR positive samples. Microscope results confirmed by qPCR were used for analysis, where qPCR was used as a reference method.

**Results:**

The sensitivity and specificity of One Step P.f/P.v Tri-line mRDTs were 96.0% (CI 93.5–97.7%) and 98.3% (CI 96.8–99.2%), respectively. One Step P.f mRDT had sensitivity and specificity of 95.2% (CI 92.5–97.1%) and 97.9% (CI 96.3–99.0%) respectively. Positive predictive value (PPV) was 97.6% (CI 95.4–98.7%) and negative predictive value (NPV) was 96.2% (CI 95.5–98.3%) for the One Step P.f/P.v Tri-line mRDTs respectively, while One Step P.f mRDT had positive predictive value (PPV) and negative predictive value (NPV) of 97.0% (CI 94.8–98.3%) and 96.7 (CI 94.9–97.9%) respectively. 9.8% (CI 7.84–11.76) of all samples tested and reported to be malaria-negative by mRDT had HRP-2 gene deletion.

**Conclusion:**

One Step Malaria P.f/P.v Tri-line and One Step Malaria P.f rapid test kits have similar sensitivity and specificity as the standard mRDT that is currently in the market, demonstrating the potential to contribute in the fight against malaria in endemic settings. However, the identified malaria parasites population with HRP-2 gene deletion pose a threat to the current mRDT usability in the field and warrants further investigations.

## Background

Malaria continues to be a major public health concern in the endemic area despite enormous efforts and funding invested in the fight against malaria to date (Tizifa et al. 2018). In 2021 world malaria report, over 240 million cases were reported in 2020 compared to 227 million malaria cases reported in 2019 (WHO 2021c). The report also showed an increase of 69,000 deaths; out of them 47,000 deaths were associated with disruption of health services due to COVID-19 pandemic (Mpina et al. 2022). The right combination of political willingness, novel technologies and effective control strategies will make the elusive target goal of reducing global malaria incidence and mortality rates by at least 90% by 2030 may be possible to achieve (WHO 2021b). In the meantime, many African countries are still struggling to maintain implementation of current expensive control measures that became ineffective in some parts of the continent and hence call for an urgent scale-up of new products, technology or implementation strategies that could drive the continent to the elimination point (Okumu et al. 2022).

Tanzania is one of the four countries reported to account for over half of all malaria deaths (4.1%) occurred globally (WHO 2021a). These statistics could be attributed to failure to reach the 2020 target for insecticide-treated nets (ITN) distribution, challenges associated with the increasing prevalence of asymptomatic cases that could not be detected by routine diagnostic methods, or due to interruption of health systems by Covid-19 pandemic (Bylicka-Szczepanowska and Korzeniewski 2022). To support malaria elimination campaign in Tanzania, different malaria vector control interventions have been deployed over the years including provision of ITN, indoor residual spraying (IRS), and targeting the breeding sites by using insecticides (Wangdi et al. 2018). However, despite these interventions, morbidity and mortality persisted in under-five children and pregnant women (Imboumy-Limoukou et al. 2020).

Diagnostic tools are, however, among the important pillars of elimination strategies as it could be used for the clinical management of the disease (Santos et al. 2020). When malaria parasite detection is done early, it facilitates early management that would prevent progression to severe disease and reduces the risk of transmitting the infection to the next person (White and Sabarwal, n.d.). Routinely, microscope and rapid diagnostic tests (RDTs) are widely used for parasite detection in both rural and urban settings. Nevertheless, each technique has its strength and weakness in terms of performance sensitivity and specificity. Malaria RDT (mRDT) is a diagnostic method that can detect parasite antigens in whole blood samples. When compared to other diagnostic techniques, it is simple to run, provides results in short time, and does not require electricity or expensive equipment (Justyna 2017).Even with the remarkable benefits of the mRDTs, batch-to-batch variations, result misinterpretation, limited sensitivity and specificity of some test kits, and the instability of stored test kits in tropical climates offer some disadvantages. Good performance of mRDTs depends on how sensitive and specific they are (Galatas et al. 2020). While several mRDTs on the current market are showing sub-optimal performance in the field, the most reliable mRDTs will be the one that can improve detection to enable effective treatment of confirmed cases to reduce complications related to severe forms of malaria.

The most sensitive mRDTs that are currently in the market detect parasite Histidine-Rich Protein 2 (HRP-2) circulating in the blood. These proteins are specific to *P. falciparum*, and expressed in abundance by asexual parasites. Previous studies conducted in the Amazon region identified patients infected with *P. falciparum* strains that had acquired deletions in the genes that encode these proteins (pfHRP-2 and pfhrp3), rendering them undetectable by HRP-2-based RDTs (Fontecha et al. 2018; Leonard et al. 2022). Since then, many studies have demonstrated the presence of such mutated strains in other countries and regions including Tanzania (Kaaya et al. 2022). The frequency and global distribution of the HRP-2 gene-deleted parasites population are not yet fully understood, although the relative incidence of these deletion mutants has been reported to threaten the usefulness of HRP-2-based RDTs (Berzosa et al. 2020; Parr et al. 2017). Screening for HRP-2 gene-deleted parasites during the field evaluation of mRDTs is critical to inform the public health official of the threat and propose an improved method for malaria diagnosis in the affected population.

An addition of new mRDT with improved performance sensitivity and specificity will merit the current battle against malaria. The InTec PRODUCT INC Company from China has introduced new mRDT kits for detecting the *Plasmodium falciparum* and other Plasmodium species. However, the field performance evaluation of these kits in terms of sensitivity and specificity was yet to be established. This study aimed to evaluate the field performance sensitivity and specificity of the new One Step Malaria (P.f/P.v) Tri-line and One Step Malaria (P.f) rapid test kits by comparing with microscopy and qPCR as reference methods.

## Methods

### Study design and population

A cross-sectional study was conducted on symptomatic participants from July 2020 to September 2021. Participants aged 5 years and above who presented to the health facilities with fever or a history of fever within 48 h or an illness that the attending doctor suspected might be due to malaria infection were targeted. Participants who met the enrollment criteria consented to participate in the study. The study involved three arms; clinical, analytical and blood type. Clinical study aimed to determine operation performance, sensitivity and specificity of the (P.f) and One Step (P.f/P.v) mRDTs test kits. A blood type study aimed to investigate the performance of independent operators when blood collected in different anticoagulants were used on the One Step Malaria (P.f) and One Step (P.f/P.v) Tri-line test kits, while the analytical study aimed to investigate whether co-infections could interfere with the performance accuracy of the One Step Malaria (P.f/P.v) and (P.f) tests.

A total of 1630 (1175 P.f negative and 455 P.f positive) patients were tested for malaria using the registered SD BIOLINE, One Step Malaria (P.f/P.v) Tri-line and One Step Malaria (P.f) rapid test kits. The current sample size calculation was based on the requirement for Technical Specifications Series for submission to WHO Prequalification Diagnostic Assessment TSS-3 Malaria rapid diagnostic tests, which requires at least 1500 participants to be enrolled for this type of analysis (Organização Mundial da Saúde 2017). The clinical study arm included 1514 samples whereby 1108 were negative and 406 positive. The analytical study included 20 negative, 20 negative with other diseases and 21 malaria positive, and blood type study included 30 negatives and 25 positives.

### Study site

The study sites were selected based on malaria transmission data obtained from the Health Management Information System (HMIS) under the national malaria control program. In the current study area, malaria transmission intensity varies within geographical region and seasonality despite that infections are acquired all year-round. The study was implemented in two districts of the Pwani region. These included the peripheral dispensaries located in Kibiti (Bungu and Mtawanya) and Bagamoyo (Fukayosi, Congo, Yombo and Mkenge) districts. Kibiti district representing stable and high malaria transmission where most cases are caused by *Plasmodium falciparum*, while few cases are caused by other *plasmodium species*. Bagamoyo district represent low malaria transmission and the transmission usually happens during and after a long period of rain. About 96% of all malaria cases are caused by *Plasmodium falciparum.* The prevalence of malaria in the general population is 13%, according to the epidemiological characterization of malaria in the area (Salim et al. 2015).

### Evaluation of malaria rapid diagnostic kits

Both direct finger prick (capillary) and whole venous blood collected in EDTA, Heparin, and Sodium citrate were parallel tested with mRDTs One Step Malaria (P.f/P.v) Tri-line and One Step Malaria (P.f) rapid test kits in the blood type study. The blood slides were examined using the World Health Organization (WHO) protocol as described elsewhere (Sumari et al. 2017). The quantitative Polymerase Chain Reaction (qPCR) were done according to method described in the previous study (Schindler et al. 2019) and both tested using only EDTA venous blood. In clinical and analytical study arms, finger prick (capillary blood) was tested with mRDTs test kits, while the reference methods (qPCR and Blood slide) were performed using EDTA venous blood.

### Quality control and data collection

Thick and thin blood smears as well as qPCR were conducted at the reference laboratory of IHI in Bagamoyo for the purpose of quality control. Laboratory scientists and technicians working at the reference laboratory were blinded to the mRDTs kits results. Results of blood slides and mRDTs were also blinded to laboratory scientists who performed qPCR. To assess inter-observer variability, mRDT results were prepared and independently interpreted by trained lay providers or trained healthcare workers and double-checked by expert laboratory technicians.

### Malaria parasite detection by qPCR

Genomic DNA was extracted from the whole blood using Quick-Gdna™ Blood MiniPrep Kit (Zymo Research, USA) following the manufactures instructions. Master Mix was prepared using Lunar Universal Probe qPCR Master Mix (New England Biolabs NEB M3004X.) as per Ifakara Health Institute laboratory protocol. The real-time qPCR assays were performed using the Bio-Rad CFX96 detection system and analysis done by using CFX Manager Software (Bio-Rad, version 3.1). *P. falciparum* genomic DNA (WHO reference from NIBSC) serially diluted into a different range of concentrations (from 100,000 parasites/microliter (p/µl) up to the lowest concentration of 0.001 p/µl) were used to generate the relative standard curves for parasite density quantification. Samples with Ct < 40 on the target gene and Ct < 35 in the housekeeping gene were considered to be positive.

### Characterization of mRDT performance on samples stratified by parasite densities

Six categories of malaria parasite density were used to characterize mRDT performance accuracy. These included: < = 10 p/µl, 11–50 p/µl, 51–100 p/µl, 101–200 p/µl, 201–2000 p/µl, and > = 2000 p/µl. The number of positives in each range was established and discrepancies were recorded to obtain the percentage agreement among the tests. Blood smear results that were only confirmed by qPCR were used as reference densities for this comparison.

### qPCR for detection of HRP-2 gene deletion

The HRP-2 gene deletion was identified from samples that were mRDTs negative but positive for microscopy and qPCR. To evaluate the effectiveness of DNA extraction and qPCR amplification, the internal control gene for pfrnr2 (PF3D7 1,015,800) with sequences of AGTATCCAAAACACTATAATTCCAAGTAC forward and ATTTTCTCCTTTCTTAACAGTTTCTTCC reverse respectively were used. Also gene deletion marker, pfrp2 (PF3D7 0,831,800) with sequences of GTATTATCCGCTGCCGTTTTTGCC forward and TCTACATGTGCTTGAGTTTCG reverse were chosen, respectively. Utilizing the Bio-Rad CFX96 detection system and the CFX Manager Software, real-time qPCR assays were performed. Master Mix was prepared and following the thermal profile was used for qPCR: 5 min at 95 ^∘^C, 45 cycles, 15 s at 95^∘^C, and 35 s at 57.5 °C.

### The impact of malaria co-infections on mRDT results

To determine the impact of malaria co-infections in the test kits, hospital guideline was used to select patients with other disease (Table 5) and were tested using both One Step Malaria (P.f/P.v) Tri-line and One Step Malaria (P.f) rapid test kits to see whether other diseases would affect the performance of the tested kits.

### Data management and statistical analysis

Data were entered in an excel database (Microsoft Excel) and used to assess clinical performance of One Step Malaria (P.f/P.v) Tri-line line and One Step Malaria (P.f) rapid test kits, as well as microscope and qPCR results from different malaria transmission intensities. The STATA software version 15 was used for establishing sensitivity and specificity of the tests.

## Results

A total of 1630 (1175 *P.f* negative and 455 *P.f* positive) patients seeking medical care and presented with fever, met the enrollment criteria at health care facilities, were tested for malaria using both the registered mRDT (SD BIOLINE), the One Step Malaria (P.f/P.v) Tri-line One Step Malaria (P.f), microscope and qPCR as reference. SD BIOLINE was used to manage patients at the health care facilities whenever needed. 62.1% (1012/1630) of participants were female and 34.5% (562/1630) were males. 10.98% (179/1630) and 23.5% (383/1630) of male participants were malaria positive and negative respectively and 15.5% (253/1630) and 46.6% (759/1630) of female participants were malaria positive and negative respectively. Gender of 3.4% (56/1630) of participants was not specified. The characteristics of the study demographic characteristics are summarized in Table 1.

**Table 1:** A summary of the study demographic characteristics

Age category	Gender	Positive	% Positive	Negative	% Negative	Subtotal number	Prevalence %
5–18 years	Male	127	59.3	87	40.7	214	32.2
	Female	146	46.9	165	53.1	311	
19–45 years	Male	45	15.9	238	84.1	283	54
	Female	97	16.2	501	83.8	598	
46–65 years	Male	7	11.7	53	88.3	60	9
	Female	6	7	80	93	86	
> 65 years	Male	0	0	5	100	5	1.3
	Female	4	23.5	13	76.5	17	
Unspecified	NA	23	41.1	33	58.9	56	3.4
Total		455		1175		1630	100

### Sensitivity and specificity of One Step Malaria RDT tests

To determine the performance of One Step Malaria (P.f/P.v) Tri-line and One Step Malaria (P.f) rapid test kits, sensitivity and specificity of the kits were calculated with reference to blood smear results that were confirmed by qPCR. The results showed that, there was no different in sensitivity and specificity of One Step Malaria (P.f/P.v) Tri-line and One Step Malaria (P.f) rapid test kits when finger prick blood were used [95.7% (93.2–97.6% CI) and 97.9% (96.3–99.0% CI)], respectively, and when venous blood were used sensitivity and specificity were [96.0% (93.5–97.7% CI) and 98.3% (96.8–99.2% CI)], respectively. Similar finding was observed for the One Step Malaria Pf test RDTs [sensitivity, 95.5% (92.9–97.3%) and specificity, 98.1% (96.6–99.1% CI)] when finger prick blood was used and [sensitivity, 95.2% (92.5–97.1% CI) and specificity, of 97.9% (96.3–99.0% CI)] when venous blood were used. Additionally, the sensitivity and specificity of SD Bioline mRDTs that are currently in the market and used in the health facilities was [94.95% (92.22–96.93% CI) and 97.75% (96.10–98.83%)] (Tables 2, 3 and 4).

**Table 2:** Sensitivity and specificity of One Step Malaria Pf/Pv Tri-line Test in reference to blood smears and qPCR

Parameter	Finger prick (%)	95% CI	Venous (%)	95% CI
Sensitivity	95.7	(93.2–97.6)	96.0	(93.5–97.7)
Specificity	97.9	(96.3–99.0)	98.3	(96.8–99.2)
PPV	97.0	(94.8–98.3)	97.6	(95.4–98.7)
NPV	97.0	(95.3–98.1)	96.2	(95.5–98.3)

**Table 3:** Sensitivity and Specificity of One Step Malaria P.f mRDTs with reference to blood smears confirmed with qPCR

Parameter	Fingerpick (%)	95% CI	Venous (%)	95% CI
Sensitivity	95.5	(92.9–97.3)	95.2	(92.5–97.1)
Specificity	98.1	(96.6–99.1)	97.9	(96.3–99.0)
PPV	97.3	(95.1–98.5)	97.0	(94.8–98.3)
NPV	96.9	(95.1–98.0)	96.7	(94.9–97.9)

**Table 4:** Sensitivity and specificity of SD Bioline mRDT with reference to blood smear confirmed with qPCR

Parameter	Value (%)	95% CI
Sensitivity	94.95	92.22–96.93
Specificity	97.75	96.10–98.83
PPV	96.75	94.44–98.12
NPV	96.48	94.65–97.70

### Performance of mRDT based on range of parasite density categorized by blood smears results confirmed by qPCR

One Step Malaria (P.f/P.v) Tri-line, One Step Malaria (P.f) rapid test kits and SD Bioline mRDTs performance were similar across the parasite density ranges (Tables 5 and 6). The Pf/Pv mRDT had slightly higher percentage agreement at parasite density range of 11–50 p/µl compared to Pf mRDT and SD Bioline mRDT (Table 5).

**Table 5:** Performance of One Step Malaria Pf/Pv Tri-line and Pf RDTs mRDT based on range of parasite density categorized by blood smears results and confirmed by qPCR

Parasite density	Pf/Pv MRDT				Pf MRDT			
	# of Pos	Agreement %	Ref	Discrepancy	# of Pos	Agreement %	Ref	Discrepancy
< 10p/ul	0	100	0	0	0	100	0	2
11–50p/ul	3	75	4	1	2	50	4	6
51–100p/l	6	50	12	6	6	50	12	4
101–200p/ul	13	76.5	17	4	12	76.5	17	4
200–2000p/ul	65	94.2	69	4	65	94.2	69	2
> 2000p/ul	273	99.6	274	1	272	99.3	274	
Grand total	360		376	16	358		376	18

**Table 6:** Performance of SD Bioline based on range of parasite density categorized by blood smears results confirmed by qPCR

Parasite density	Number of positive	Reference method	% of agreement	Discrepancy
< 10 p/µl	0	0	100	0
11–50 p/µl	2	4	50	2
51–100 p/µl	6	12	50	6
101–1000 p/µl	13	17	76.5	4
1001–2000 p/µl	65	69	94.2	4
> 2000 p/µl	271	274	98.9	3
Grand total	357	376		19

### The proportional of samples with Histidine-Rich Protein 2 (HRP-2) gene deletion

Three hundred and seventy-six samples were malaria positive by both blood slides and qPCR. 44 out of 376 samples that were positive by both qPCR and microscopy were negative for One Step Pf/Pv Tri-line, One Step Malaria Pf only and SD Bioline mRDTs and were further analyzed for HRP-2 gene deletion by qPCR assays. 7/44 (15.9%) samples gave positive results for HRP-2 gene, implying that the gene was there. However, 37/44 (84.1%) were negative for the HRP-2-gene. The general proportion of HRP-2 gene deletion reported from the current study was 9.8% (7.84–11.76) (Table 7). The mean parasite densities for these samples in blood slide and qPCR were 1975.1 p/µl and 3219.9 p/µl respectively.

**Table 7:** Proportion of Histidine-Rich Protein 2 gene deletion on samples confirmed to have Pf positive by blood smear and qPCR

	qPCR + /BS +	qPCR + /BS + /RDT-	qPCR + /BS + /RDT-/, No HRP-2 gene deletion	qPCR + /BS + /RDT-/with HRP-2 gene deletion	Proportion of HRP-2 gene deletion (95% CI)
Number of participants	376	44	7	37	9.8% [7.84–11.76]
Mean parasite density for blood slide = 1975.1			Mean parasite density for qPCR = 3219.9		

**Fig. 1:**
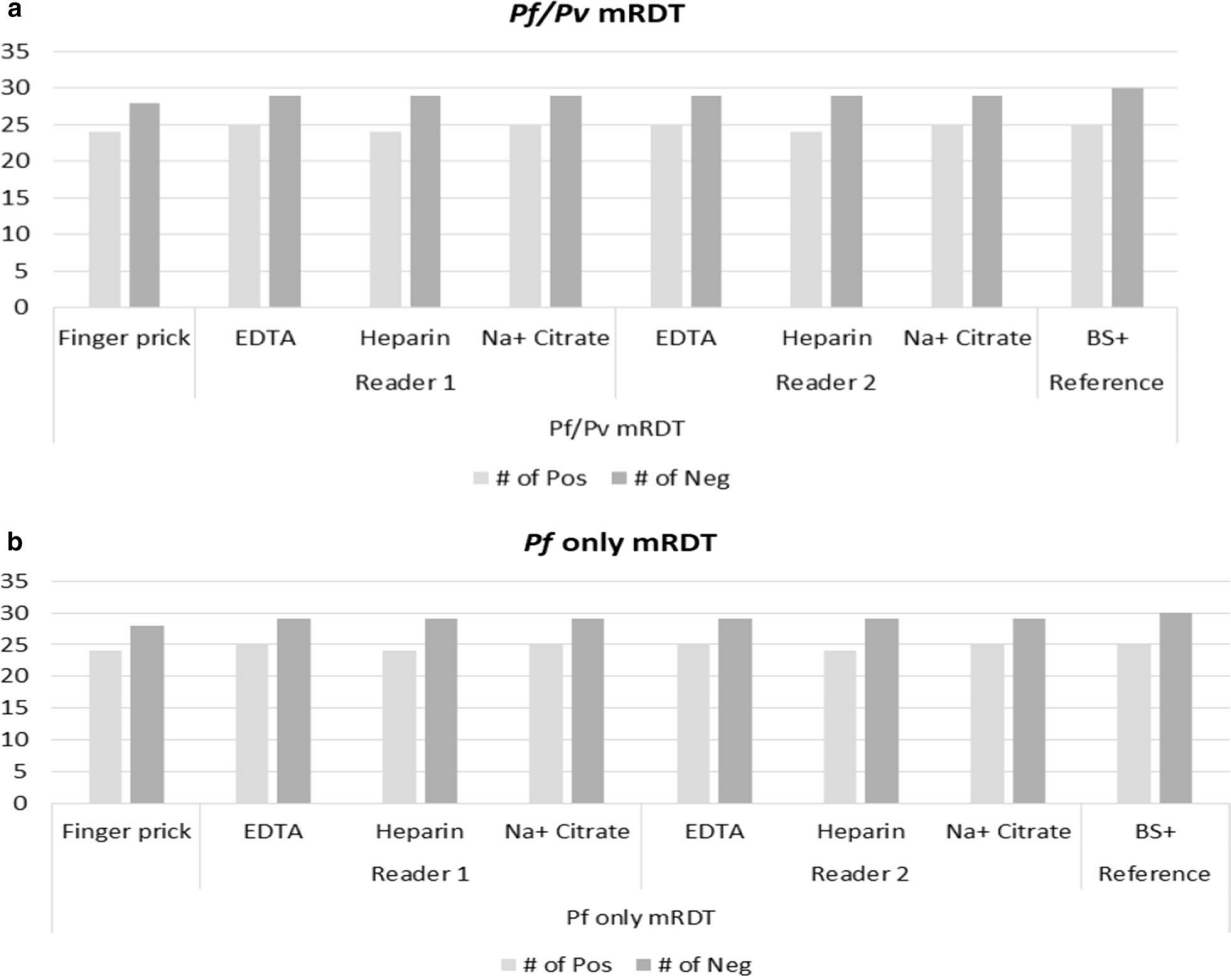
**a** Histograms representing number of positive and negative results for One Step Malaria Pf/Pv Tri-line mRDT when blood collected from different anticoagulants were used for testing using different operators. **b** Histograms representing number of positive and negative results for One Step Malaria Pf RDTs when blood collected from different anticoagulants were used for testing using different operators

We further investigated whether anticoagulant may have an effect of mRDT results. By using two independent readers, we found no different in number of positives or negatives detection when either of anticoagulant is used to collect blood for mRDT testing, Fig. 1a for (P.f/P.v) Tri-line mRDT and 1b (P.f) mRDT.

Next, we examined the impact of other co-infections on mRDT results. The results showed that One Step Malaria (P.f/P.v) Tri-line and One Step (P.f) mRDT could detect all cases detected by blood smears and qPCR, when blood collected from juvenile patients who had co-infected with other diseases were tested. Twenty-eight (28) patients who had malaria positive results as detected by both BS/qPCR and RDTs were also positive by other diseases infections (Table 8).

**Table 8:** Performance of the One Step Pf/Pv mRDTs Tri-line and one step P.f tests in relation to diseases other than malaria and malaria confection

Disease	mRDT positive	mRDT negative	Microscopy positive	Microscopy negative	Total
Upper respiratory tract infection	8	6	8	6	14
Urinary tract infection	8	0	8	0	8
Tonsillitis	0	1	0	1	1
Dental caries	1	0	1	0	1
Diarrhea	2	1	2	1	3
Enteric fever	1	1	1	1	2
Pneumonia	3	0	3	0	3
HIV	3	1	3	1	4
Tuberculosis	2	0	2	0	2

## Discussion

The current study demonstrated that the sensitivity and specificity of the new One Step Malaria (P.f/P.v) Tri-line and (P.f) mRDTs were similar to that of the standard kit (SD Bioline) in the field evaluation. The sensitivity and specificity of other novel mRDT have also been compared to the standard mRDT kits (SD Bioline) which have been to the market for decade (Willie et al. 2018). The results of the current study are in agreement with the results obtained from other mRDTs that are currently in the market (Slater et al. 2015). Interestingly, One Step Malaria (P.f/P.v) Tri-line mRDTs detected up to 50% of samples with parasite density as lower as 11parasites/µl and over 75% of samples in parasite ranges of 101–200 parasites/µl similar to standard mRDT kit (SD Bioline).

Thin and thick blood slides remained as gold standard for malaria diagnosis to date (Mathison and Pritt 2017) (Belachew et al. 2022). The rapid diagnostic tests (RDTs) and microscope are the common tests that are routinely used for the management of malaria cases in Tanzania, although microscope remained to be the gold standard [11] [12]. Malaria diagnostic tools with sufficient limit of detection to identify even the asymptomatic and reservoir with low parasitemia but enough to transmit will supplement the existing malaria control strategies (Mbabazi et al. 2015). Despite the fact that qPCR is more sensitive, this technique has limited usefulness in the standard care, hence is mainly used for confirmation of malaria parasite species after diagnosis is established by rapid diagnostic tests or blood slides (Global Malaria Programme, 2017). The results from this study showed that both One Step Malaria (P.f/P.v) Tri-line and One step (P.f) test kits have capability of detecting proportion of cases with very low parasite density (~ 11parasites/µl). The results are in agreement with the most mRDT that are currently in the market which have reported to have capacity to detect correctly parasite density of below 100 p/µl (Mwesigwa et al. 2019; Wu et al. 2015)(Taylor et al. 2019).

The performance accuracy of the mRDT could be influenced by the deletion of HRP-2. Histidine-Rich Protein-2 (HRP-2) is produced in abundance by *Plasmodium falciparum* and it is highly stable (Kong et al. 2021). In this study, we determined the presence of HRP-2 gene deletion in samples that was tested negative by mRDTs but positive by blood slide and qPCR, where 9.8% of all *P. falciparum*-positive samples had HRP-2 gene-deleted which causes false negative in some participants. According to WHO if estimated HRP-2/3 gene deletion is higher than 5%, then there is a high confidence that the proportion of false negative mRDT results in symptomatic *P. falciparum* patients is caused by pfHRP-2/3 deletion (WHO 2020). One possibility of the high number of HRP-2 gene deletion detected in this study could be awareness to population to be tested for malaria before treatment or self-medication. Since most of the testing is done by mRDT, there is possibility of selection pressure to occur and leave the HRP-2 gene deletion parasite populations in the community (Agaba et al. 2019). Other studies have reported similar finding of presence of HRP-2 gene deletion parasites in the community (Golassa et al. 2020; Koita et al. 2012; Thomson et al. 2019) that lead to false negative mRDT results.

Furthermore, blood collected in different anticoagulants did not affect the performance of One Step Malaria (P.f/P.v) Tri-line and One Step (P.f) mRDTs test kits as compared to standard mRDT kits (SD Bioline). The scored sensitivity and specificity were similar to sensitivity and specificity reported by other mRDT kits available in the market (Charpentier et al. 2020; Eticha et al. 2020; O et al. 2021). In addition, the samples collected from patients who had malaria co-infected with other diseases did not affect the performance of the test kit (One Step Malaria (P.f/P.v) Tri-line and One Step Malaria (P.f) tests).

### Study limitation

First, the current One Step Malaria (P.f/P.v) Tri-line and One Step Malaria (P.f) mRDTs test kits were designated to detect antibody for *P.falciparum* and *P. vivax* species only and was not designated to detect patients presented with fevers caused by *P. malariae* and *P. ovale and P. knowles*. This might have led to misidentification of etiology of non-falciparum and non-vivax fever. Moreover, the current study did not analyzed antibodies against pfHRP-2 by Enzyme Linked Immunosorbent Assay (ELISA) to determine whether there was presence of HRP-2 proteins in individuals who had HRP-2 gene deletion. Although the qPCR itself is sufficient to confirm gene deletion, the results from antibodies could have been a positive supplement for the finding. Lastly, the detection of other co-infection was sorely depended on clinical judgments made by clinicians using national treatment algorithms and not laboratory testing.

## Conclusion

The current study demonstrated that the sensitivity and specificity of novel One Step Malaria (P.f/P.v) Tri-line and One Step Malaria (P.f) mRDTs test kits were similar to that of SD Bioline which is the standard kit used routinely in the health care facilities in Tanzania and elsewhere. The proportional of samples with HRP-2 gene deletion reported in the study population and causing false negative mRDT results warrants further studies to map the prevalence of gene deletion and determine how it affects the current malaria control strategies.

## Data Availability

Data set will be made available on reasonable request from the corresponding author.

## Abbreviations

*Pf*: *Plasmodium falciparum*
*Pv*: *Plasmodium vivax*
HRP-2: Histidine-rich protein 2
mRDTs: Malaria rapid diagnostic tests
qPCR: Quantitative polymerase chain reaction
WHO: World Health Organization
EDTA: Ethylenediaminetetraacetic acid
IHI: Ifakara Health Institute
DNA: Deoxyribonucleic acid
NIBSC: National Institute for Biological Standards and Control
NA: Non applicable
PPV: Positive predictive value
NPV: Negative predictive value
